# Alterations in amygdala–prefrontal circuits in infants exposed to prenatal maternal depression

**DOI:** 10.1038/tp.2016.146

**Published:** 2016-11-01

**Authors:** J Posner, J Cha, A K Roy, B S Peterson, R Bansal, H C Gustafsson, E Raffanello, J Gingrich, C Monk

**Affiliations:** 1 Department of Psychiatry, Columbia College of Physicians and Surgeons, New York, NY, USA; 2 New York State Psychiatric Institute, New York, NY, USA; 3 Department of Psychology, Fordham University, New York, NY, USA; 4 Institute for the Developing Mind, Children's Hospital Los Angeles and the Keck School of Medicine, University of Southern California, Los Angeles, CA, USA

## Abstract

Prenatal exposure to maternal depression is common and puts offspring at risk for developing a range of neuropsychiatric disorders. Despite its prevalence and adverse associations, neurobiological processes by which prenatal maternal depression (PMD) confers risk remain poorly understood. Maternal mood and fetal behavior were assessed between 34 and 37 gestational weeks. Using resting-state functional magnetic resonance imaging (fMRI) and diffusion MRI, we examined functional and structural connectivity within amygdala–prefrontal circuits in 64 infants (mean age=5.8±1.7 weeks) with (*n*=20) and without (*n*=44) *in utero* exposure to PMD. Resting fMRI and diffusion MRI both indicated atypical amygdala–prefrontal connectivity in PMD-exposed infants: Resting fMRI indicated increased inverse, or negative, functional connectivity between the amygdala and the dorsal prefrontal cortex (PFC), bilaterally, and diffusion MRI indicated decreased structural connectivity between the right amygdala and the right ventral PFC. Spectral dynamic causal modeling supported these findings suggesting altered amygdala–PFC effective (or directed) connectivity in PMD-exposed infants. Last, path analyses supported a mechanistic account relating PMD to a third-trimester fetal behavior: PMD alters amygdala–PFC connectivity, which in turn, is associated with an increase in fetal heart rate reactivity to *in utero* perturbation. These data suggest that the maturation and coordination of central and peripheral physiology are altered by prenatal exposure to maternal depression. To the best of our knowledge, this is the first study to directly associate infant MRI measures with a behavior—fetal heart rate response, and supports hypotheses that PMD-associated variations in the development of amygdala–PFC circuits are relevant for future neurobehavioral maturation.

## Introduction

Prenatal exposure to maternal depression is common with 10–15% of pregnant women in the US experiencing a major depressive episode.^1^ The exposure puts the offspring at risk for developing a range of neuropsychiatric disorders.^2^ Beyond the role of shared genes, the neurobiological processes by which prenatal maternal depression (PMD) influences children's developmental trajectories are poorly understood.

Recent magnetic resonance imaging (MRI) studies have begun to characterize neurobiological correlates of exposure to PMD. In one of the first, children of mothers with PMD showed cortical thinning, particularly over the frontal lobes.^3^ This is consistent with research suggesting that cortical thinning may be a risk marker for depression.^4^ A second study found that the microstructure of the amygdala, indexed by fractional anisotropy, may be altered in newborns of mothers with PMD.^5^ A third study showed that PMD-exposed infants had increased functional connectivity between the amygdala and several frontal regions.^6^ Together these results suggest that exposure to PMD may influence the development of the frontal cortex, and, particularly, amygdala–prefrontal circuits, with implications for future affect regulation. For example, altered amygdala–prefrontal connectivity has been implicated in pediatric depression,^7, 8^ with hypothesized effects on affect regulation.^9^

Our study aimed to further investigate the potential role of PMD in children's neurobehavioral development in three ways. First, we examined PMD effects on neural connectivity in infants shortly after birth. Prior research assessed infants no earlier than 6 months of age, limiting the ability to disentangle PMD from the effects of postpartum depression. Second, we directly tested associations between amygdala–prefrontal connectivity and a fetal behavior. Building on our prior work^10^ and that of others,^11, 12^ we studied fetal heart rate (FHR) reactivity to *in utero* stimuli, initiated via maternal autonomic nervous system responses to acute laboratory stress. Heightened FHR responses are associated with PMD exposure and a more reactive infant temperament.^13^ Establishing that amygdala–prefrontal circuits—developed over the course of gestation though tested shortly after birth—are associated with a third-trimester fetal behavior reflective of amygdala–prefrontal cortex (PFC) circuits to the brainstem^14, 15, 16^ would provide functional significance of the variation in brain development and its relevance for future social–emotional trajectories. Third, we used two complementary, but distinct, MRI indices of neural connectivity (functional and structural connectivity obtained from resting fMRI and diffusion MRI, respectively), coupled with computational modeling. Combining MRI modalities and modeling techniques provides a comprehensive view of brain circuit development assessed shortly following birth.

We obtained resting fMRI and diffusion MRI scans from a total of 64 infants with (*n*=20) and without (*n*=44) *in utero* exposure to PMD. Our study included assessment of maternal affective symptoms and a measure of fetal behavior. On the basis of prior literature, we hypothesized that infants exposed to PMD would show altered functional and structural connectivity within amygdala–prefrontal circuits, though the novelty of this research precluded specific hypotheses about the direction of these effects. We then conducted exploratory analyses to understand further the associations between PMD and the maturation of amygdala–prefrontal circuits. First, using spectral dynamic causal modeling (sDCM), we estimated the direction of neural signaling (that is, effective connectivity) between the amygdala and the PFC. This permitted exploring whether reciprocal amygdala–PFC connections, which are detectable in adults, also are evident in young infants, and whether these reciprocal connections are affected by PMD. Second, we tested associations between amygdala–PFC connectivity and FHR reactivity. Last, we used path analysis to examine associations between PMD, FHR reactivity and amygdala–PFC connectivity.

## Materials and methods

### Participants

Healthy pregnant women, ages 18–45, were recruited at Columbia University Medical Center. Women were excluded if they acknowledged smoking or use of recreational drugs, lacked fluency in English or were multiparous. Additional descriptions of the participants, as well as comparisons of screened versus included participants, are provided in the Supplementary Materials. The participants provided informed consent; New York State Psychiatric Institute Institutional Review Board approved all the procedures.

### Study procedures

Between 34 and 37 weeks' gestation (mean gestation=36.3±2.0 weeks), pregnant women were assessed for mood symptoms and then underwent fetal assessment. Within weeks of birth (mean age at scan=5.8±1.7 weeks, Table 1), sleeping, nonsedated infants underwent a multimodal MRI session.

### Infant characteristics

Gestational age at the fetal assessment session (see the 'Fetal Assessment' subsection) was determined by ultrasound examinations and last reported menstrual cycle. Gestational age at birth and birth weight were determined from the medical record.

### Maternal mood symptoms

The participants completed the self-reported Center for Epidemiological Studies Depression scale (CES-D)^17^ and the Perceived Stress Scale,^18^ as well as the interview-based Hamilton Rating Scale for Depression.^19^ Depression group (PMD) versus not depressed group (N-PMD) was based on a cutoff of ⩾16 on the CES-D, as described elsewhere.^20, 21^ To assess psychiatric disorders in addition to depression, the schedule for affective disorders and schizophrenia was used for sensitivity analyses (see Supplementary Analyses). Missing the schedule for affective disorders and schizophrenia was due to participant time constraints. There were no differences in subjects who completed the schedule for affective disorders and schizophrenia versus those who did not (*P*-values >0.3).

### Fetal assessment

Participants were in a semi-recumbent position for 25 minutes as fetal movement and heart rate were acquired. Data were obtained using a Toitu MT 325 fetal actocardiograph (Toitu, Tokyo, Japan), which detects fetal movement and heart rate via a single transabdominal Doppler transducer. Fetal data were collected from the Toitu's output port, digitized at 50 Hz using a 16-bit A/D card and analyzed offline. Fetal measures were obtained while women rested quietly for five minutes (baseline), then completed a 5-minute computerized Stroop color-word task (stressor), followed by a 5-minute recovery period, a breathing task and a final, 5-minutes recovery period. Within-subject FHR reactivity scores were computed by subtracting the mean and standard deviation of the FHR during the stressor period from baseline values, as previously described.^22^

### MRI scanning

Images were acquired on a GE Signa 3T whole-body scanner with an eight-channel head coil. Acquisition of a three-dimensional spoiled gradient recall image was followed by echoplanar images (TR=2200 ms, TE=30 ms, 90° flip angle, single excitation per image, slice thickness 3.5 mm, 19 × 19 cm field of view, 64 × 64 matrix, 24 slices) with an effective resolution of 3.75 × 3.75 × 3.5 mm and whole-brain coverage. Five runs of 102 volumes (3 min and 44.4 s) were obtained for each participant. Two diffusion MRI runs were acquired for each infant with parameters: TR 8000 ms; slice thickness 2 mm; voxel size 2 × 2 × 2  mm^3^; slices 50. The series included three initial images acquired without diffusion weighting and with diffusion weighting along 11 noncollinear directions (*b*=600  s m^−2^).

### Head motion during scanning

We took several steps to limit the influence of in-scanner head motion. First, we excluded any fMRI run with a peak Framewise Displacement (FD) over 1 mm.^23^ Second, we matched the numbers of fMRI runs across subjects by selecting two of the five total runs with the least head motion (all the participants had two usable runs except for two participants having only one). These strict criteria resulted in minimal head motion in both groups (PMD group: mean peak FD=0.20±0.190 mm, range=0.03 mm~0.62 mm; N-PMD group: mean peak FD=0.13±0.095 mm, range=0.03 mm~0.41 mm). Two infants showed peak FD values >0.5 mm. Third, we censored frames with excessive motion. Additional details regarding head motion and censored frames are provided in the Supplementary Materials. We then estimated an additional head motion parameter, DVARS.^23^ No group differences in FD or DVARS were detected (*P*-values >0.5). Head motion parameters were included as nuisance regressors in both the subject- and group-level analyses (see the subsections 'Resting State Image Processing' and 'Statistical Analyses').

For diffusion MRI scans, we estimated four head motion parameters: average translation, average rotation, percentage of slices with signal dropout and signal dropout severity.^24^ No scans were excluded because of excessive in-scanner head motion (mean rotation in PMD group=0.61±0.200 mm; mean rotation in N-PMD group=0.57±0.127 mm; mean translation in PMD group=0.005±0.0022° mean translation in N-PMD group=0.004±0.0015°). Group comparisons of head motion estimates are provided in the Supplementary Materials. Head motion parameters were included as nuisance regressors for group-level analyses (see the subsection 'Statistical Analyses').

### Resting-state image processing

Image preprocessing was performed with SPM8 and CONN toolbox.^25^ Images were motion-corrected, coregistered with an anatomical scan, normalized to an infant template brain,^26^ resampled at 2 mm and smoothed with a Gaussian kernel of 6 mm full width at half maximum. Temporal band-pass filtering (0.008–0.09 Hz) was applied. Nuisance regressors included six head motion parameters, and orthogonal fMRI time series (extracted using the component-based noise correction (CompCor)^27^ method), and their derivatives, within each individual's white matter and cerebrospinal fluid. The preprocessing stream using CompCor, combined with band-pass filtering and modeling of movement parameters, provides sensitivity and specificity to detect unbiased correlations in resting-state networks.^28, 29^

### Seed-based functional connectivity

Resting fMRI time series were correlated region of interest (ROI) by voxel for each infant. Fisher-z transformation was applied. Connectivity maps were generated with the seed ROI of the amygdala (each hemisphere separately) derived from a published infant atlas.^26^

### Diffusion MRI tractography

Complete details for tractography processing are in the Supplementary Materials. Briefly, diffusion MRI data were preprocessed with the FSL 5.0 Diffusion Toolbox with probtrackx2.^30^ Masks for tractography were the same as those used for resting fMRI analyses, warped into individual structural space using nonlinear transformation (FNIRT) and transformed into the individual diffusion space using linear registration (FLIRT). Streamlines were generated via Markov Chain Monte Carlo sampling procedure. Since the accuracy of Markov Chain Monte Carlo sampling depends on the number of iterations, we optimized our tractography solutions by increasing the number of iterations from the default of 5000 to 25 000. We used an amygdala structural atlas^26^ as the seed mask; the cerebrospinal fluid, segmented from an infant's structural scan, was the stopping mask. Finally, using classification masks, we calculated the probability that the amygdala connected with the PFC target regions (24 regions; Supplementary Materials contains ROIs) relative to the overall streamlines from the amygdala, defined as following:^31^


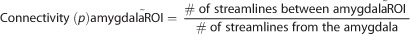


Therefore, our structural connectivity measures represent relative, region-to-region, connection probability. Target regions whose probabilistic tract measures failed to reach a threshold of 0.02% of the total estimated streamlines were excluded.^31, 32, 33, 34^ Diffusion MRI analysis was performed on a high performance computing cluster at Columbia University Medical Center.

### Statistical analyses

We entered the seed-based connectivity maps into group-level factorial models with Group as the single factor with two levels (PMD and N-PMD). We restricted analysis to prefrontal ROIs (see Supplementary Materials). Head motion parameters, infant age at scan, sex and birth weight were included as nuisance regressors. For diffusion MRI tractography, we used multivariate general linear models containing amygdala–prefrontal structural connectivity measures as the dependent variable and the same nuisance regressors except for the diffusion tensor imaging head motion parameters.

We corrected for multiple statistical comparisons using a cluster extent method. A minimum cluster extent to meet a corrected alpha <0.05 given a voxel-wise *P* of 0.005 was estimated using 3dClustim in AFNI.^35^ Both whole-brain (using whole-brain mask) and prefrontal ROI (using prefrontal mask) analyses were conducted. For diffusion MRI analyses, multivariate general linear model results were corrected using the false discovery rate (FDR).^36^

### Exploratory analyses: sDCM

On the basis of group differences in amygdala–PFC functional connectivity (see the 'Results' section), we assessed effective connectivity between the amygdala, dorsal PFC and ventral PFC using sDCM (DCM12, revision 6080). This approach addressed two questions: (i) are reciprocal amygdala–prefrontal connections evident in the neonatal brain, as described in adults?^37^ and (ii) if so, are these connections influenced by PMD? Further details on effective connectivity and sDCM methodology are provided in the Supplementary Materials.

### Clinical and behavioral correlations

We considered brain-behavior associations by examining the associations between amygdala–PFC connections and fetal behavior while adjusting for maternal group, infant age at scan, sex and birth weight. We tested the associations via robust linear regression to curtail the influence of potential outliers.

### Path analysis

We performed path analysis to explore the interrelationships between PMD, amygdala–PFC connectivity and fetal behavior. For this analysis, PMD (indexed by CES-D) served as the independent variable, connectivity (indexed by the left amygdala–dorsal PFC functional connectivity) as the mediator, and FHR reactivity as the dependent variable. An indirect, mediation effect was tested using bootstrapping (5000 iteration), which does not assume a normal distribution of mediation effects. Mediation analyses were performed using AMOS 23 (SPSS, IBM, Armonk, NY, USA).

## Results

### Descriptive information

Pregnant women were between 20 and 43 years old (mean=29.22±6.01; Table 1). MRI data were collected in a total of 92 infants. Of these, usable MRI data were obtained in a total of 64 infants. Twenty-eight infants were excluded from the MRI analyses for technical reasons including the infant not tolerating the MRI scan, excessive head motion and/or imaging artifacts. Of the 64 infants who had usable MRI data, this consisted of *n*=20 infants exposed to PMD and *n*=44 infants not exposed to PMD (N-PMD). Of these 64 infants, resting fMRI analyses included 48 infants (*n*=17 PMD infants and *n*=31 N-PMD infants). Diffusion MRI analyses included 49 infants (*n*=18 PMD infants and *n*=31 N-PMD infants). FHR reactivity was assessed in 39 infants (*n*=13 PMD infants and *n*=26 N-PMD infants).

The infants with and without usable MRI data were comparable on demographics and anthropomorphics (*P*-values >0.3, see Supplementary Materials). The PMD and N-PMD groups did not differ significantly on infant birth weight, infant age at MRI scan, infant sex, maternal age or maternal education (Table 1).

### Voxel-wise seed-based functional connectivity

**Figure 1:**
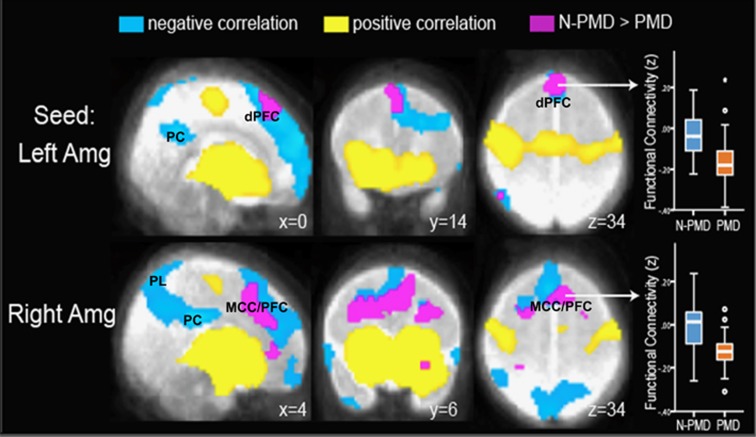
Infants exposed to prenatal maternal depression (PMD) show increased inverse, or negative, functional connectivity between the amygdala and the dorsal prefrontal cortex. Seed-based connectivity maps are presented with seeds in the left and right amygdala (Amg). Positive and negative correlations (that is, positive and inverse connectivity) are presented in yellow and blue, respectively. Infants exposed to prenatal maternal depression (PMD) and infants unexposed to N-PMD both display inverse connectivity between the left and right amygdala and the dorsal prefrontal cortex (dPFC), posterior cingulate cortex (PC) and parietal lobes (PL). Regions in purple/fuchsia indicate differences in connectivity strength in N-PMD versus PMD infants. Box plots (right column) demonstrate that relative to N-PMD infants, exposed infants (PMD) show increased inverse connectivity between the left amygdala and the dPFC and between the right amygdala and the midcingulate cortex/prefrontal cortex (MCC/PFC).

In both the groups, seed-based connectivity maps generated from the left and right amygdala showed significant positive connectivity with the ventral PFC, ventral anterior cingulate, hippocampus and contralateral temporal lobe (Supplementary Figure 3). Significant inverse, or negative, connectivity was detected in both the groups between the amygdala and the dorsal PFC, dorsal anterior and posterior cingulate cortices, and parietal lobes (Figure 1). In the PMD, compared with N-PMD group, there was significantly greater inverse functional connectivity between the right amygdala and a large cluster spanning the midcingulate cortex and dorsal PFC across the left and right hemispheres (whole-brain corrected *P*<0.001; peak *z*=3.43, MNI_x,y,z_=[−2 11 24], cluster size=3294 mm^3^ at uncorrected *P*=0.005 and full width at half maximum of 9.63 mm; Figure 1 and Supplementary Table 1) and between the left amygdala and a large cluster within the dorsal PFC spanning the left and right hemispheres (prefrontal ROI corrected *P*<0.02; peak *z*=3.53, MNI_x,y,z_=[4 21 37], cluster size=1041 mm^3^ at uncorrected *P*=0.005 and full width at half maximum of 9.63 mm; Supplementary Table 1). Analysis with uncensored volumes showed comparable results (Supplementary Table 2A). There were no regions in which PMD infants showed reduced inverse functional connectivity. No group differences were detected in positive functional connectivity.

### Diffusion tractography

**Figure 2:**
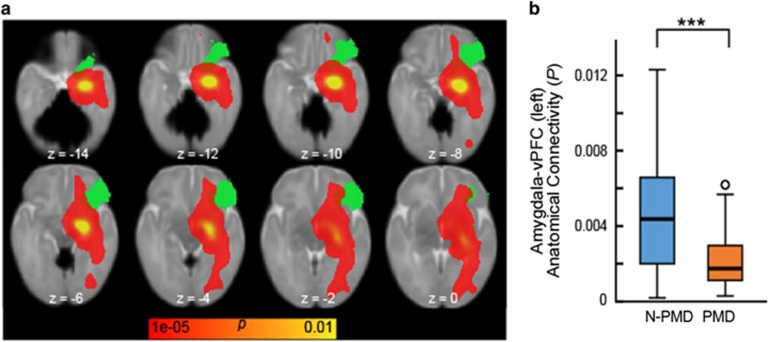
Infants exposed to prenatal maternal depression (PMD) show reduced structural connectivity between the amygdala and the ventral prefrontal cortex. (**a**) A probabilistic structural connectivity map (shown in red–yellow) overlaid with a ventral prefrontal cortex (vPFC) mask. White matter tracts from the amygdala were estimated using probabilistic diffusion tractography; amygdala–PFC structural connectivity was then calculated on the basis of the ratio of numbers of tracts reaching each PFC target region relative to all estimated amygdala tracts. For presentational purpose, the estimated tracts were registered and overlaid with the standard neonatal brain. (**b**) A box plot of amygdala–vPFC structural connectivity. General linear model revealed a significant decrease in amygdala–vPFC structural connectivity in infants exposed to PMD relative to unexposed infants (N-PMD). ***P<0.001.

Compared with the N-PMD group, PMD infants showed decreased structural connectivity between the right amygdala and right ventral PFC (*P*_FDR_=0.0219; Figure 2). In a confirmatory analysis using Network-Based Statistics to consider amygdala-seeded connectivity across the whole brain, only the right amygdala–right ventral PFC connection showed a significant association with PMD (*P*_FWE_<0.05).

### Spectral dynamic causal modeling

**Figure 3:**
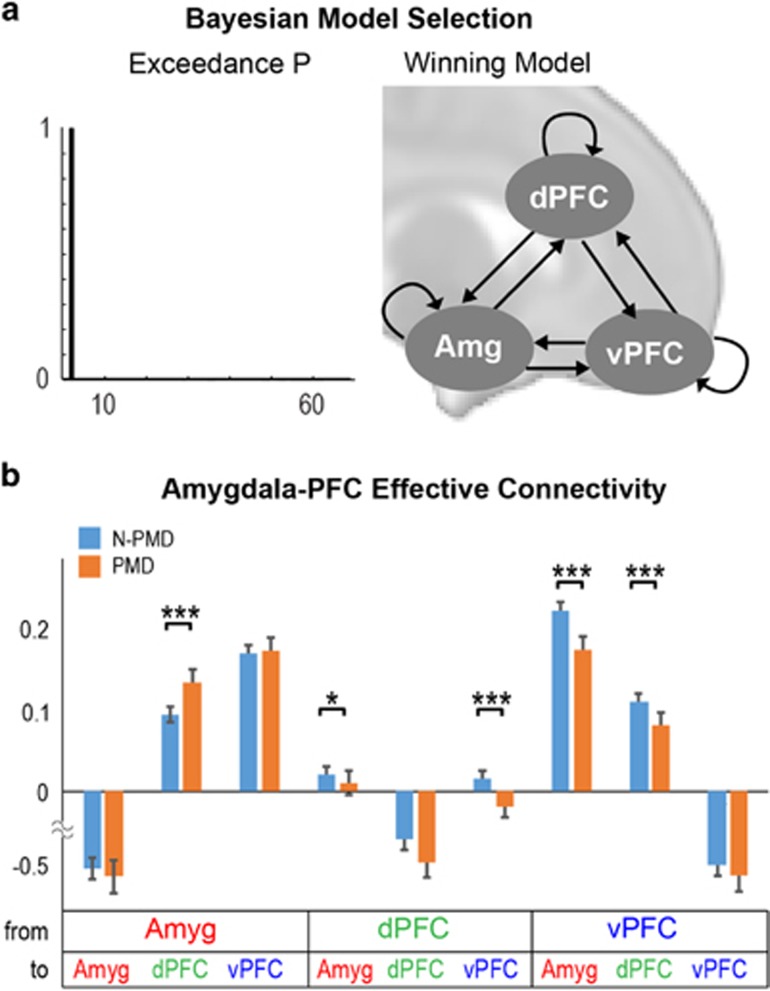
Spectral dynamic causal modeling suggests effective connectivity in the infant brain between the amygdala, dorsal prefrontal cortex and ventral prefrontal cortex. (**a**) Bayesian Model Selection (BMS) showed strong evidence (exceedance probability) for a fully interconnected model with reciprocal connections between the amygdala, dorsal prefrontal cortex (dPFC) and ventral prefrontal cortex (vPFC) across all the subjects (model shown in the right panel). Separate BMS within each group showed identical results (exceedance probability >0.99). (**b**) Effective connectivity estimates were obtained from Bayesian model averaging in each group. Relative to infants unexposed to prenatal maternal depression (N-PMD), infants exposed to prenatal maternal depression (PMD) displayed increased effective connectivity from the amygdala to the dPFC; and decreased effective connectivity from the dPFC and vPFC to the amygdala, dPFC to vPFC and vPFC to dPFC (two-tailed *t*-tests; false discovery rate corrected for multiple comparison). *P<0.05; ***P<0.001.

Using Bayesian model selection, we tested whether the infant brain displays an interconnected neural network between the amygdala, ventral PFC and dorsal PFC. We considered all possible combinations of a three-node network (total of 64 models). A fully interconnected network was selected as the best model with an exceedance probability of 1 (Figure 3a). The PMD and N-PMD groups tested separately did not change this finding.

We next tested whether PMD influenced effective connectivity (that is, directed connectivity) within the tripartite amygdala–PFC network. Relative to unexposed infants, those exposed to PMD displayed: (i) increased bottom–up excitatory influence from the amygdala to the dorsal PFC; (ii) decreased top–down cortical excitatory influences from both the dorsal and ventral PFC to the amygdala; and (iii) decreased reciprocal intercortical connections between the dorsal and ventral PFC (Figure 3b).

### Behavioral correlations

Greater FHR reactivity in response to a mild maternal stressor correlated with greater inverse functional connectivity between the left amygdala and the dorsal PFC (*t*=−3.58, *P*=0.001, robust regression; Supplementary Figure 4A) and with greater excitatory influence from the amygdala to the ventral PFC (*t*=4.15, *P*=0.0004, robust regression; Supplementary Figure 4B). No significant outliers were detected (mean Cook's distance=0.05±0.098, min/max=0.00005/0.45). Effects adjusted for maternal group, infant age at scan, sex and birth weight.

### Path analysis

**Figure 4:**
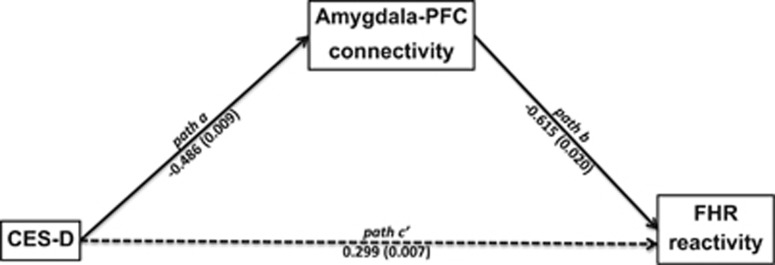
Path analysis suggests that increased inverse amygdala–prefrontal functional connectivity is a potential mechanism by which maternal distress during pregnancy enhances fetal heart rate reactivity. In the first linear regression of this path analysis, we found that maternal depressive symptoms (as measured by the Center for Epidemiological Studies Depression scale (CES-D)) was a significant predictor of connectivity between the left amygdala and the left dorsal prefrontal cortex (Amygdala–PFC, path a, beta=−0.486, *P*=0.009). In the second linear regression model, we found that while controlling for CES-D, Amygdala–PFC connectivity was a significant predictor of fetal heart rate reactivity (FHR reactivity, path b, beta=−0.615, *P*=0.020). Bias-corrected bootstrapping confirmed the significance of the indirect effect of CES-D on FHR reactivity (path c', coefficient=0.299; *P*=0.007).

Path analysis supported a mechanistic account that PMD is associated with greater FHR reactivity through its effects on amygdala–PFC connectivity (Figure 4). The mediation model showed a significant indirect, mediation effect of maternal depression (indexed by CES-D) on FHR reactivity through amygdala–PFC connectivity (indexed by the left amygdala–dorsal PFC functional connectivity; standardized mediation effect=0.299, *P*=0.007, Bias-corrected percentile method based on bootstrap samples). A significant association was found between CES-D and amygdala–dorsal PFC functional connectivity (standardized effect=−0.486, *P*=0.009) and between amygdala–dorsal PFC functional connectivity and FHR reactivity (standardized effect=−0.615, *P*=0.020).

### Supplementary analyses

Additional sensitivity and covariate analyses are described in the Supplementary Materials.

## Discussion

We examined the influence of PMD on infant functional and structural connectivity within amygdala–PFC circuits using resting fMRI and diffusion MRI, and computational modeling. Resting fMRI and diffusion MRI both pointed to atypical amygdala–PFC connectivity in PMD-exposed infants: Resting fMRI indicated increased inverse, or negative, functional connectivity between the amygdala and the dorsal PFC, bilaterally, and diffusion MRI indicated decreased structural connectivity between the right amygdala and the right ventral PFC. The sDCM supported these findings suggesting altered amygdala–PFC effective connectivity in PMD-exposed infants, characterized, in part, by increased bottom–up excitatory influence from the amygdala to the dorsal PFC. Using path analysis, we found evidence for a mediating pathway relating PMD, amygdala–PFC connectivity and a behavior, third-trimester FHR response: PMD exposure is associated with increased amygdala–PFC inverse connectivity, which is associated with greater FHR responses to *in utero* perturbation resulting from maternal autonomic nervous system stress reactivity. Collectively, these findings highlight the influence of PMD on neural maturation and the potential role of this exposure on future neurobehavioral development.

Inversely correlated fMRI signal between the amygdala and the dorsal PFC has been reported in prior resting fMRI studies of adults and children.^38, 39^ For example, in a cross-sectional study, functional connectivity between the amygdala and dorsal PFC was found to be inverse across development from early childhood to adulthood.^39^ We found that compared with N-PMD infants, those with PMD exposure showed significantly stronger amygdala–dorsal PFC inverse functional connectivity. One interpretation is that PMD represents an adverse *in utero* exposure that accelerates neurodevelopment, rending this circuit more mature in PMD infants. Accelerated development may prepare the fetus for a concordant, challenging postnatal environment, potentially encountered as maternal postpartum depression and/or the psychosocial factors that contributed to maternal perinatal mood disturbance. Analogous findings of precocious amygdala–PFC connectivity have been reported in children institutionalized during infancy,^40^ suggesting priming of this circuit in response to developmental stressors.^40^ Early adversity has been associated with accelerated sexual maturation, advanced postnatal motor and cognitive development, and greater neural integration at birth based on auditory evoked responses.^41, 42^ According to the Predictive Adaptive Response model, whether this priming poses a risk for future psychopathology may, in part, depend on the ‘match' with the psychosocial circumstances of the postnatal environment.^43^

Although we postulate that PMD accelerates amygdala–dorsal PFC development, we did not find evidence for this effect in amygdala–ventral PFC circuits. Probabilistic tractography showed that PMD infants have reduced structural connectivity between the right amygdala and the right ventral PFC. Spectral DMC offered convergent evidence suggesting reduced top–down signaling from the right ventral PFC to the right amygdala in PMD infants. Diffusion MRI research in adults suggests that reduced structural connectivity within the amygdala–ventral PFC circuit is associated with greater trait anxiety.^44^ Likewise, resting fMRI studies point to reduced amygdala–ventral PFC connectivity in adults with depression.^45, 46^ Given the putative role of amygdala–ventral PFC circuits in extinguishing conditioned fears,^47^ disruption of this circuit may render PMD infants less adept at extinguishing fear responses. Behavioral assessments over subsequent developmental stages could test this hypothesis.^48^ We cannot say with certainty why PMD potentially disrupts amygdala–ventral PFC circuits, while accelerating development of amygdala–dorsal PFC circuits; however, this may relate to the divergent functions, developmental trajectories and/or stress sensitivity of these circuits.

The sDCM analyses suggest that infants, regardless of PMD exposure, have an integrated amygdala–PFC circuit. Similar to the architecture of this circuit in adults, reciprocal connections were detected between the amygdala, dorsal PFC and ventral PFC. This surprising finding suggests that amygdala–PFC topology is already formed by early infancy. Its early maturation perhaps arises from the circuit's phylogenetic importance following the adage that ontogeny recapitulates phylogeny.

We believe this is the first study to directly associate infant MRI measures with fetal behavior. We found that increased amygdala–dorsal PFC inverse functional connectivity was associated with FHR increases to changes in the prenatal environment. Path analysis supported a mediation effect such that maternal distress over the course of pregnancy may increase inverse amygdala–dorsal PFC functional connectivity which, in turn, is associated with upregulation in FHR responses to perturbations arising from acute maternal stress. Animal studies support this mechanistic account. For example, electrolytic lesions to the central nucleus of the amygdala blunt normal cardiovascular responses to conditioned stimuli.^15^ The amygdala is thought to modulate cardiovascular responses to affective stimuli via amygdalofugal and amygdalopetal projections to the brainstem.^15^ It is possible that over the course of gestation, exposure to maternal distress shapes amygdala–PFC circuits, which in turn, contribute to a more advanced physiological response to stimuli in the fetus. This is evidenced in the FHR increase to perturbation whereas fetuses of healthy, euthymic women have been shown not to have a FHR change during this paradigm.^10^ Coordination of central and peripheral physiological responses to stimuli may prepare the fetus for a postnatal environment in which adversity is anticipated.

Study limitations should be considered. First, we cannot disentangle genetic versus environmental contributions to amygdala–PFC development. Nonetheless, altered amygdala–PFC development may represent an important biological variant in infants at risk for psychopathology. Second, our mediation results should be considered provisional because of the temporal sequence in which the mediator (connectivity) was measured after the outcome (fetal behavior). This limitation is difficult to circumvent without the availability of fetal MRI measures. Third, we do not know whether PMD effects on amygdala–PFC development predict subsequent childhood behaviors. Longitudinal research could address this question.^48^ Fourth, we cannot fully exclude potential effects of infants' exposure to women's postpartum depressive symptoms. However, controlling for postpartum depressive symptoms did not alter hypothesis testing (Supplementary Tables 2A and 4B). Moreover, the mean age of the infants was <6 weeks and thus postpartum effects on brain connectivity should be small relative to the *in utero* exposure. Fifth, women in the PMD and N-PMD groups differed in their family incomes and presence of comorbid disorders. Adjusting for these factors did not alter our results, but their confounding influence cannot be entirely excluded. It is also possible that income may serve as a marker of other significant factors influencing brain development such as the quality of maternal diet.^49^ Sixth, our diffusion MRI data were collected with 11 gradient directions. A high number of gradient directions may be preferable for tractography. Finally, though ours is the largest MRI study to examine PMD effects in early infancy, replication in a larger sample is warranted.

Familial risk for psychiatric illness has long been observed. Increasingly, this risk has been understood as having developmental origins that are potentially modifiable. Here, we push back the time frame to identify variation in perinatal brain-behavior functioning related to *in utero* exposure to maternal depression, before significant postnatal influences and relevant to future affective regulation. These findings suggest a potential for very early identification of risk phenotypes for the purposes of primary intervention, as well as the possibility that familial risk for psychopathology occurs, in part, through the transmission of pregnant women's psychiatric symptoms to their children.

## Figures and Tables

**Table 1:** ** **Demographic and clinical characteristics of study participants** **

	*Controls (*n=*44)*	*Depressed (*n=*20)*	*Test statistic (df)*	P*-value*
Age at scan (weeks)	5.99±1.86	5.66±1.74	*t* (62)=0.685	0.496
Gestational age at birth (weeks)	39.30±1.07	39.23±1.02	*t* (62)=0.199	0.843
				
*Sex*			*X*^2^(1)=1.772	0.183
Male	21	6		
Female	23	14		
				
Infant birth weight (g)	3278.27±476.65	3347.58±395.86	*t* (61)=−0.556	0.580
Maternal age	30.85±5.66	28.61±6.66	*t* (62)=1.387	0.170
Maternal education (years)	15.33±3.09	14.15±4.40	*t* (27.81)=1.083	0.288
				
*Maternal race/ethnicity*			Fisher's exact=0.663	0.909
Hispanic/Latina	24	10		
White/Caucasian	10	4		
Black/African American	5	3		
Other	5	3		
				
*Total family income*			Fisher's exact=10.972	0.009
$0–25 000	11	12		
$26 000–50 000	11	1		
$51 000–100 000	15	2		
$100 001+	7	5		
				
Primary diagnosis on SADS	*n*=1 PTSD *n*=7 missing SADS	*n*=3 MDD *n*=14 DD NOS *n*=3 missing SADS		
Maternal PSS	17.55±7.29	28.62±8.01	*t* (61)=−5.44	<0.001
Maternal HAM-D	4.83±3.59	12.48±8.74	*t* (59)=−4.87	<0.001
Maternal CES-D	7.24±4.26	21.04±10.50	*t* (62)=−7.51	<0.001
Comorbid DSM-IV psychiatric disorder by SADS	1*	5^a^	Fisher's exact=7.394	0.024

Abbreviations: CES-D, Center for Epidemiological Studies Depression scale; DD NOS, depressive disorder not otherwise specified; HAM-D, Hamilton Rating Scale for Depression; MDD, major depressive disorder; PSS, perceived stress scale; SADS, schedule for affective disorders and schizophrenia.

a Bipolar disorder, anxiety not otherwise specified, panic disorder (with and without agoraphobia), specific phobia, obsessive compulsive disorder.
