# Supplementary material for: Alterations in amygdala–prefrontal circuits in infants exposed to prenatal maternal depression
Source: Transl Psychiatry. 2016 Nov 1;6(11):e935–. doi: 10.1038/tp.2016.146 (PMC5314110; doi:10.1038/tp.2016.146)
Supplement: Supplementary Information [file tp2016146x1.docx]

**Supplemental Methods**

**Study Sample**:

A total of 964 women were approached about the study and 178 were eligible and agreed to participate (reasons for non­–enrollment were: 9 women not in age range; 84 delivered before prenatal session; 151 high risk pregnancy/medical condition; 68 lost to follow up; 35 moved location; 125 not interested in any research; 131 not interested in MRI session; 39 not able to make time commitment; 34 repeated no show to prenatal session; 57 non-English speaker; 53 no longer pregnant by first session (miscarriage, termination)). There were no differences in participants’ age, race/ethnicity, prenatal CESD scores, infant birth weight or gestational age at birth between those enrolled (178) versus those not (786). Of the 178 enrolled, 92 participated in infant MRI sessions due to the following reasons: 9 declined MRI session, 4 withdrew, 12 scanner equipment problem, 13 lost to follow up, 13 dropped due to medical complications, 14 scheduling logistics issues, 21 investigators’ decision not to scan as a robust number of infants already in N–PMD group relative to expense of scanning.

The 92 enrollees who had infant MRI scans were comparable to the 178 total enrollees. We found only two differences: the 178 total enrollees had infants with slightly less weight at birth (3269/ 424.47 SD vs 3398/478.70 SD, p = .05) and had one Native American participant, while those not in the MRI study had 0. Otherwise, no differences were detected on race/ethnicity, CESD, mom age, gestational age at birth, or income.

Of 92 infants with MRI scans, 64 infants had usable MRI data. The remaining 28 infants (92–64) were excluded from MRI analyses for technical reasons including the infant not tolerating the MRI scan, excessive head motion, and/or imaging artifacts. Of 64 infants who had usable MRI data, this consisted of n=20 infants exposed to prenatal maternal depression (PMD) and n=44 infants not exposed to prenatal maternal depression (N-PMD). Of these 64 infants, the resting fMRI analyses included 48 infants (n=17 PDM infants and n=31 N-PMD infants). The DTI analyses included 49 infants (n=18 PDM infants and n=31 N-PMD infants). Fetal heart rate (FHR) reactivity was assessed in 39 infants (n=13 PDM infants and n=26 N-PMD infants).

Comparisons for the infant with and without usable MRI data showed that they were comparable on gestational age at birth (p=0.6), birth weight (p=0.3), maternal age (p=0.4), and maternal level of depression (p=0.3), as indexed by the Center for Epidemiological Studies Depression scale (CES–D) ([1](#_ENREF_1)).

**Effective Connectivity and Spectral Dynamic Causal Modeling (sDCM)**

Stephan et al. ([3](#_ENREF_3)) define effective connectivity as “describ(ing) the causal influences that neural units exert over another.” This is distinct from “functional connectivity” which refers to temporal correlations in neural activity without inferences of causal or directed interactions. We used spectral dynamic causal modeling (sDCM) to estimate effective connectivity within amygdala-prefrontal circuits.

Spectral DCM was performed in the following four steps: (i) extraction of BOLD fMRI time series from each subject using individual coordinates based on anatomical and/or functional criteria; (ii) specification of the model space; (iii) model estimation; and (iv) model comparison. First, fMRI time series (the first eigenvectors) was extracted from the amygdala, dPFC, and vPFC from preprocessed, temporally filtered, despiked, detrended BOLD time series (processed through CONN toolbox pipeline routine). For the amygdala, we used the same anatomical atlas used in resting state fMRI analysis for consistency. For the dPFC, within the group difference map of amygdala-seeded FC (thresholded at voxel-wise *P* of 0.005) within the anatomical atlas of dPFC, times series was extracted from a sphere of 4 mm radius centered on the peak coordinates of amygdala-seeded functional connectivity in each subject. For the vPFC, timeseries were similarly extracted. The coordinates within which the timeseries was extracted were defined by the vPFC region showing peak functional connectivity with the amygdala. This was not constrained by group differences in amygdala-vPFC functional connectivity because we did not detect significant group differences in amygdala-vPFC connectivity.

Second, for model specification, we considered the entire model space. All the possible combinations of between-nodes-connections were considered, while the recurrent connection of each node was fixed to exist (“A matrix” in a DCM term). Third, DCMs were estimated using second-order statistics characterizing spectral densities over frequencies–cross spectra–based on Fourier transform of the cross correlation of the time series. Details of the spDCM procedure are described in elsewhere.([4](#_ENREF_4))

­Lastly, we examined two different aspects of the amygdalo-prefrontal circuit–model space and model parameters ([5](#_ENREF_5)). These two kinds of inferences require different sequences of analysis in DCM. For inferences on model space, we tested whether the amygdalo-prefrontal circuit would exist in the neonatal brains. To this end, we performed random effects BMS ([6](#_ENREF_6)) across all subjects, within the N-PMD group, and within the PMD group. On the other hand, for inferences on model parameters, we investigated whether PMD would influence effective connectivity. To this end, we performed Bayesian Model Averaging (BMA) in each group separately to generate group-specific parameters, which were then compared across groups using t-tests. Results were corrected for multiple comparisons using False Discovery Rate.

**Head Motion during Scanning**

As recommended elsewhere ([7](#_ENREF_7)), we examined the potential confounding influence of head motion to the fMRI-based connectivity measures and dMRI-tractography. For fMRI, we calculated the root mean square and framewise displacement; for dMRI, we used average translation, average rotation ([8](#_ENREF_8)). Non-parametric Kolmogorov-Smirnov tests showed a significant group difference in average rotation of dMRI (*P* = 0.02), but not in any other parameters (*P*’s > 0.59) (Supplement Figure 1 and 2). Of note, all reported effects were based on models containing the motion parameters as nuisance covariates.

For resting fMRI data, we censored volumes with peak FD > 0.5mm (as well as neighboring volumes). Two infants had frames that met this threshold. For one subject 26 (12.7%) out of 204 volumes (178 volumes; 6 min 31.6 sec) were detected as outliers; for the other, 11 (5.4%) out of 204 volumes (193 volumes; 7 min 4.2 sec). Results in the main text indicate fMRI analyses excluding censored volumes. Results without censoring volumes were similar and are presented in Supplemental Table 2A.

Two of the infants in the study had only 1 usable resting fMRI run and thus arguably may not have had sufficient resting fMRI data to robustly estimate connectivity. Hypothesis testing remained significant after excluding the two infants with single runs (Supplemental Table 2A).

**Definition of Prefrontal Target Regions of Interest**

Amygdala-seeded structural connectivity was estimated across 12 prefrontal regions derived from a published atlas of the neonatal brains ([9](#_ENREF_9)): the orbitofrontal cortex (superior), middle frontal gyrus, orbitofrontal cortex (middle), inferior frontal gyrus (opercular), inferior frontal gyrus (triangular), orbitofrontal cortex (inferior), olfactory, superior frontal gyrus (medial), orbitofrontal cortex (medial), rectus, anterior cingulate gyrus, and middle cingulate gyrus. Structural connectivity was derived from the left and right amygdala seeds, respectively; only ipsilateral connections were considered.

**Supplementary Analyses**

We conducted several supplementary analyses to determine if our hypothesis testing was confounded. These sensitivity analyses did not meaningfully impact the results (direction of effects and significance) of hypothesis testing. The functional and structural connectivity results from the sensitivity analyses are presented in Supplemental Table 2A and 2B, respectively.

We also adjusted our models for post-partum depressive symptoms. Hypothesis testing remained significant (Supplemental Table 2A and 2B).

Lastly, we adjusted our models for group differences in the incomes of the mothers using hierarchical regression (standard linear regression was unstable due to multicollinearity). We confirmed that when adding income to our models accounted for non-significant variance of our amygdala-PFC functional connectivity estimates.

**Supplemental Tables**

**Supplemental Table 1.** Effects of PMD on Amygdala-PFC Resting State Functional Connectivity.

| **seed** | **name** | **cluster p (corrected)** | **cluster extents (mm^3^)^†^** | **peak Z** | **MNI** | | |
| --- | --- | --- | --- | --- | --- | --- | --- |
|  |  |  |  |  | x | y | z |
| **Right amygdala** | |  |  |  |  |  |  |
| Contrast: N-PMD > PMD | |  |  |  |  |  |  |
| Analysis: Whole Brain | |  |  |  |  |  |  |
|  | MCC/PFC | <0.001 | 3294 | 3.43 | -2 | 11 | 24 |
|  |  |  |  | 3.40 | 5 | 14 | 22 |
| **Left amygdala** |  |  |  |  |  |  |  |
| Contrast: N-PMD > PMD | |  |  |  |  |  |  |
| Analysis: Prefrontal ROI | | |  |  |  |  |  |
|  | dPFC | <0.02 | 1041 | 3.53 | 4 | 21 | 37 |
|  |  |  |  | 2.83 | -6 | 20 | 39 |
| * Volumes with peak FD > 0.5mm were censored; † at P < 0.005, FWHM = 9.63 mm (estimated using SPM) | | | | | | | |

**Supplemental Table 2A. Supplementary Analyses for Resting fMRI Data**

| seed | name | cluster p (corrected) | cluster extents (mm3)* | peak Z | MNI | | |
| --- | --- | --- | --- | --- | --- | --- | --- |
|  |  |  |  |  | x | y | z |
| **Right amygdala** | | | | | | | |
| Contrast: N-PMD > PMD | | | | | | | |
| **Analysis 1:** Excluding 3 participants prenatally exposed to SSRIs (N=45) | | | | | | | |
|  | MCC/PFC | WB^a^: <0.001 | 6098 | 3.94 | 5 | 14 | 22 |
|  |  |  |  | 3.83 | 0 | 12 | 22 |
| **Analysis 2:** Excluding 5 moms with comorbidity (N=43) | | | | | | | |
|  | MCC/PFC | WB^a^: <0.02 | 1937 | 3.59 | 20 | 0 | 27 |
|  |  |  | 934 | 3.33 | -1 | 7 | 27 |
|  |  |  | 894 | 3.18 | -12 | 2 | 24 |
| **Analysis 3:** Excluding 9 moms without SADS (N=38) | | | | | | | |
|  | MCC/PFC | ROI: <0.03 | 894 | 3.24 | -19 | 6 | 18 |
| **Analysis 4:** Excluding 2 infants with only 1 resting fMRI run (N=46) | | | | | | | |
|  | MCC/PFC | WB^a^: <0.005 | 2711 | 3.33 | 5 | 14 | 22 |
|  |  |  |  | 3.2 | -2 | 12 | 22 |
| **Analysis 5:** Censored fMRI frames *not* excluded (N=48) | | | | | | | |
|  | MCC/PFC | WB^a^: <0.04 | 1000 | 3.70 | 21 | 2 | 23 |
| **Analysis 6**: Adjusting for post-natal maternal CES-D (N=48) | | | | | | | |
|  |  | WB^a^: <0.02 | 2152 | 3.65 | 1 | 24 | 6 |
| **Left amygdala** | |  |  |  |  |  |  |
| Contrast: N-PMD > PMD | | |  |  |  |  |  |
| **Analysis1:** Excluding 3 SSRI treated moms (N = 45) | | | | | | | |
|  | dPFC | ROI^b^: <0.01 | 1173 | 3.88 | 3 | 18 | 33 |
| **Analysis2:** Excluding 5 moms with comorbidity (N = 43) | | | | | | | |
|  | dPFC | ROI: <0.02 | 927 | 3.69 | 4 | 21 | 38 |
| **Analysis 3:** Excluding 9 moms without SADS (N = 38) | | | | | | | |
|  | dPFC | ROI: <0.02 | 1115 | 3.76 | 1 | 17 | 35 |
| **Analysis 4:** Excluding 2 infants with only 1 resting fMRI run (N=46) | | | | | | | |
|  | dPFC | ROI: <0.02 | 1133 | 3.61 | 1 | 16 | 36 |
|  |  |  |  | 3.1 | -2 | 18 | 33 |
| **Analysis 5:** Censored fMRI frames *not* excluded (N=48) | | | | | | | |
|  | dPFC | ROI: <0.02 | 1197 | 3.66 | 4 | 21 | 37 |
| **Analysis 6**: Adjusting for post-natal maternal CES-D (N=48) | | | | | | | |
|  | dPFC | ROI: <0.02 | 1085 | 3.48 | -1 | 17 | 32 |
| *at P < 0.005, FWHM xyz = 9.63 mm (estimated in SPM); a, WB, Whole brain correction; b, ROI correction. | | | | | | | |

**Supplemental Table 2B. Supplementary Analyses for Diffusion MRI Data**

|  | Group | | 95% Wald Confidence Interval | | Hypothesis Test | |
| --- | --- | --- | --- | --- | --- | --- |
|  | B | Std. Error | Lower | Upper | Wald Chi-Square | P-Value |
| **Analysis 1:** Excluding 3 participants prenatally exposed to SSRIs (n=46) | 0.0027 | 0.0008 | 0.0012 | 0.0042 | 11.9874 | 0.0005 |
| **Analysis 2**: Excluding 4 pregnant women with comorbidity (n=45) | 0.0025 | 0.0010 | 0.0005 | 0.0044 | 6.3176 | 0.0120 |
| **Analysis 3**: Excluding 8 pregnant women without SADS (n=41) | 0.0030 | 0.0213 | 0.0015 | 0.0046 | 15.4731 | <0.0001 |
| **Analysis 4:** Adjusting for post-natal maternal CES-D (N=49) | 0.003 | 0.008 | 0.001 | 0.004 | 13.143 | 0.0002 |

**Supplemental Figures**

**Supplemental Figure 1.** **Boxplots of peak framewise displacement of resting state fMRI of each group.** Numbers above the columns denote significance of non-parametric Kolmogorov-Smirnov test (2-tailed).


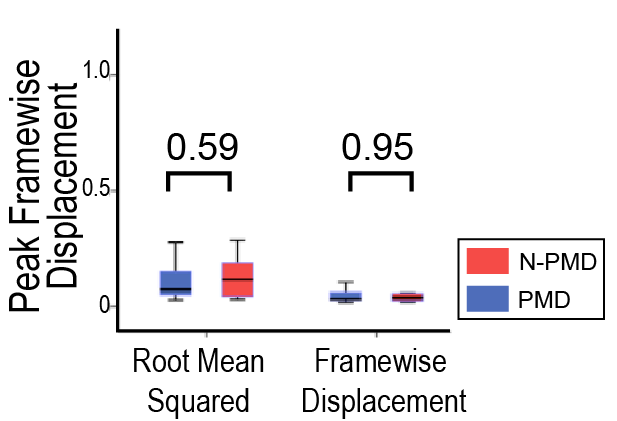


**Supplemental Figure 2.** **Boxplots of head motion parameters of diffusion MRI of each group.** Numbers above the columns denote significance of non-parametric Kolmogorov-Smirnov test (2-tailed).


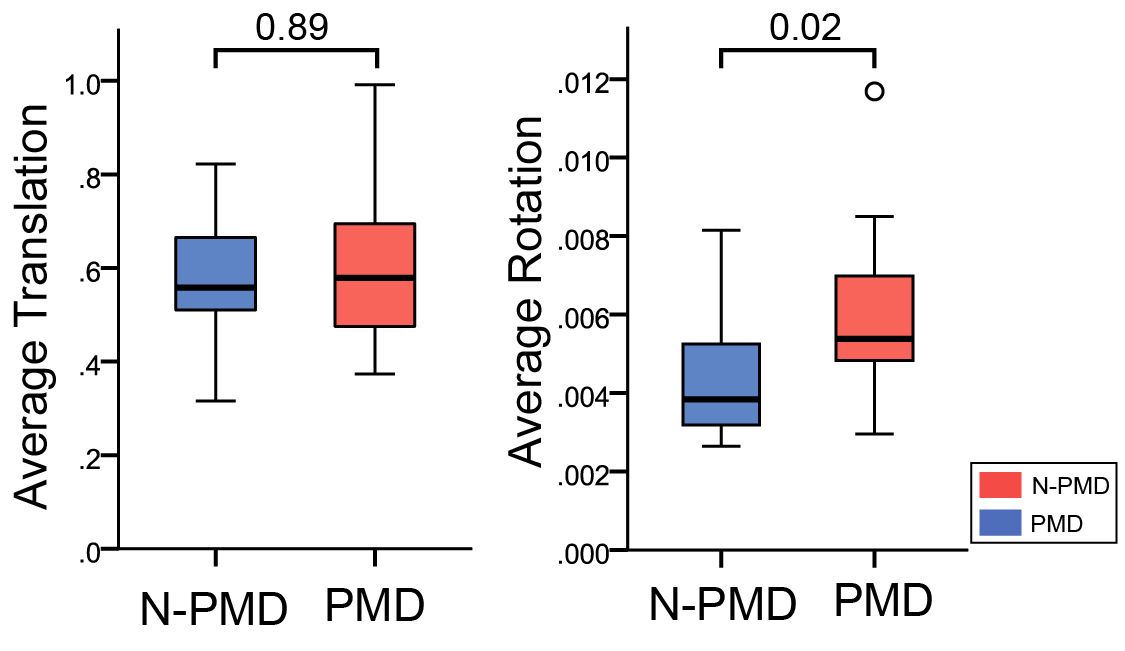


**Supplemental Figure 3.** **Positive amygdala functional connectivity across groups.** Voxel-wise analyses of resting-state fMRI showed similar patterns of amygdala functional connectivity across groups. Activation maps shown were thresholded at uncorrected P of 0.005.


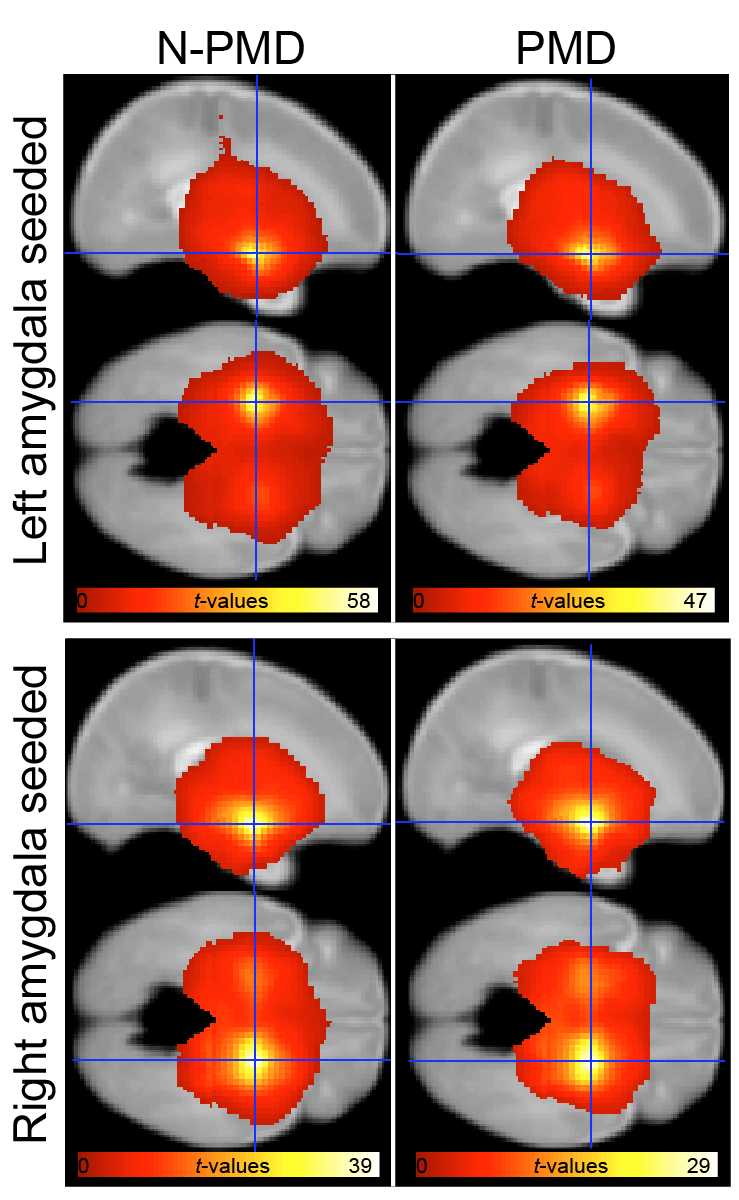


**Supplemental Figure 4. Scatterplots indicating significant associations between infants’ amygdala–PFC connectivity and fetal heart rate reactivity during a mild maternal stressor**. **A**, Greater inverse, or negative, functional connectivity (FC) between the left amygdala and the dorsal prefrontal cortex (dPFC) correlated with greater fetal heart rate reactivity. **B**, Greater effective connectivity (EC) from the amygdala to the ventral prefrontal cortex (vPFC) correlated with greater fetal heart rate reactivity.

**
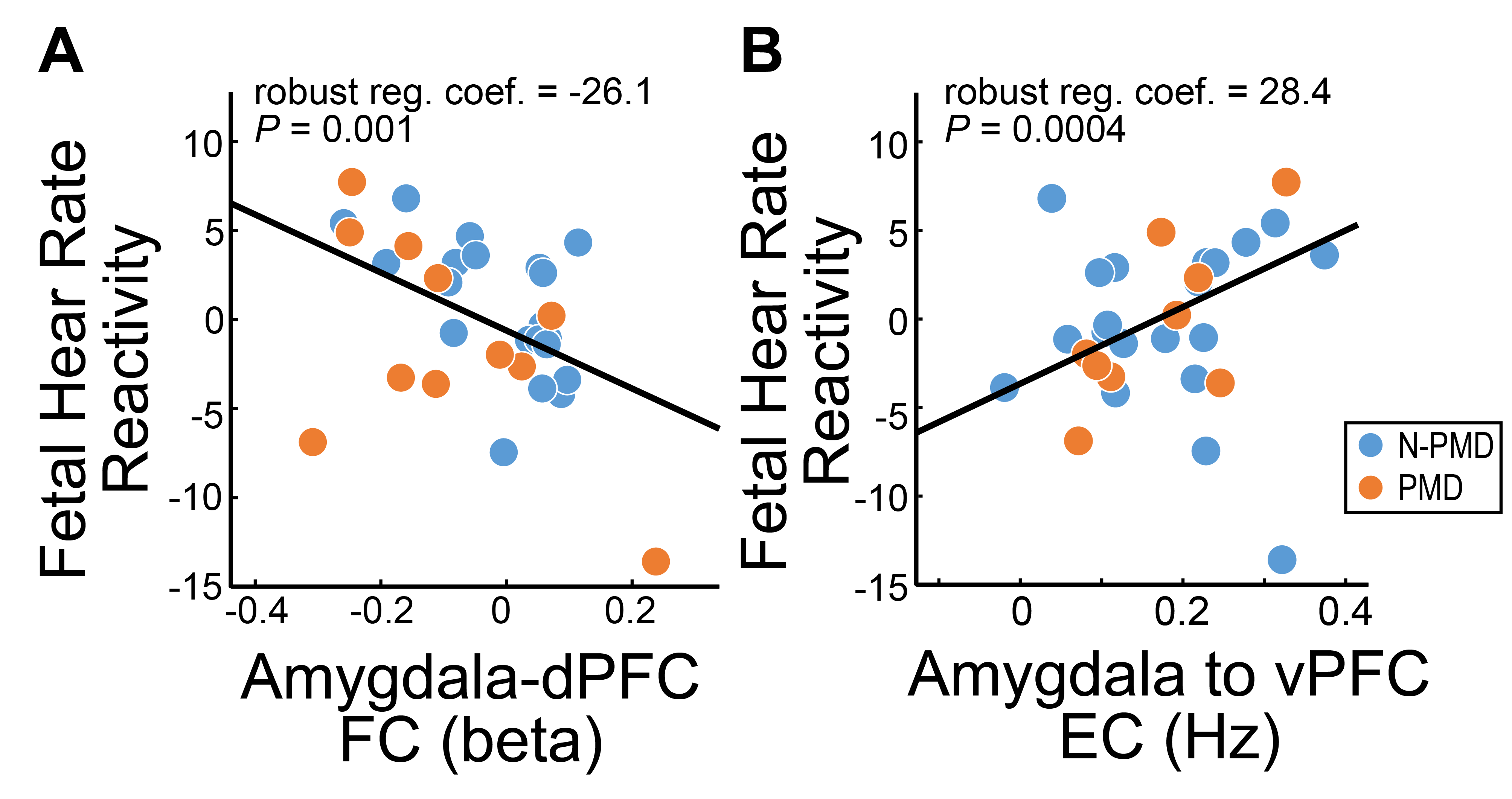
**

**Supplemental References**

1. Radloff LS. The CES-D scale a self-report depression scale for research in the general population. Applied psychological measurement. 1977;1(3):385-401.

2. Monk C, Myers MM, Sloan RP, Werner L, Jeon J, Tager F, et al. Fetal heart rate reactivity differs by women's psychiatric status: an early marker for developmental risk? Journal of the American Academy of Child and Adolescent Psychiatry. 2004;43(3):283-90.

3. Stephan KE, Friston KJ. Analyzing effective connectivity with functional magnetic resonance imaging. Wiley Interdisciplinary Reviews: Cognitive Science. 2010;1(3):446-59.

4. Friston KJ, Kahan J, Biswal B, Razi A. A DCM for resting state fMRI. Neuroimage. 2014 Jul;94:396-407. PubMed PMID: 24345387. Pubmed Central PMCID: PMC4073651. eng.

5. Stephan KE, Penny WD, Moran RJ, den Ouden HE, Daunizeau J, Friston KJ. Ten simple rules for dynamic causal modeling. Neuroimage. 2010 Feb 15;49(4):3099-109. PubMed PMID: 19914382. Pubmed Central PMCID: 2825373.

6. Stephan KE, Penny WD, Daunizeau J, Moran RJ, Friston KJ. Bayesian model selection for group studies. Neuroimage. 2009 Jul 15;46(4):1004-17. PubMed PMID: 19306932. Pubmed Central PMCID: 2703732. Epub 2009/03/25. eng.

7. Power JD, Barnes KA, Snyder AZ, Schlaggar BL, Petersen SE. Spurious but systematic correlations in functional connectivity MRI networks arise from subject motion. Neuroimage. 2011.

8. Yendiki A, Koldewyn K, Kakunoori S, Kanwisher N, Fischl B. Spurious group differences due to head motion in a diffusion MRI study. Neuroimage. 2014;88:79-90.

9. Shi F, Yap PT, Wu G, Jia H, Gilmore JH, Lin W, et al. Infant brain atlases from neonates to 1- and 2-year-olds. PLoS One. 2011;6(4):e18746. PubMed PMID: 21533194. Pubmed Central PMCID: PMC3077403. eng.
